# Host‐microbe interaction in the gastrointestinal tract

**DOI:** 10.1111/1462-2920.13926

**Published:** 2017-11-10

**Authors:** Aimée Parker, Melissa A. E. Lawson, Laura Vaux, Carmen Pin

**Affiliations:** ^1^ Quadram Institute Bioscience Norwich Research Park NR4 7UA UK

## Abstract

The gastrointestinal tract is a highly complex organ in which multiple dynamic physiological processes are tightly coordinated while interacting with a dense and extremely diverse microbial population. From establishment in early life, through to host‐microbe symbiosis in adulthood, the gut microbiota plays a vital role in our development and health. The effect of the microbiota on gut development and physiology is highlighted by anatomical and functional changes in germ‐free mice, affecting the gut epithelium, immune system and enteric nervous system. Microbial colonisation promotes competent innate and acquired mucosal immune systems, epithelial renewal, barrier integrity, and mucosal vascularisation and innervation. Interacting or shared signalling pathways across different physiological systems of the gut could explain how all these changes are coordinated during postnatal colonisation, or after the introduction of microbiota into germ‐free models. The application of cell‐based *in‐vitro* experimental systems and mathematical modelling can shed light on the molecular and signalling pathways which regulate the development and maintenance of homeostasis in the gut and beyond.

## Introduction

Our gut is home to a large and complex community of microorganisms termed the intestinal microbiota. The dynamic environment within the intestine presents a challenge to both the host and the intestinal microbiota to maintain a mutualistic relationship throughout life (Tannock, 2007). In this review, we focus on the factors which influence bacterial composition throughout the gastrointestinal tract, and on the cross‐talk between the microbiota and the host at the gastrointestinal (GI) barrier, which results in the development of a precise GI organisation and functionality. We discuss this in the context of what is currently known about gut microbiota interaction with host defences, and research tools and models that can be used to study these interactions.

Following pioneering experiments in clinical and animal models over a century ago (Cushing and Livingood, 1900) researchers have generated a variety of tools, including animal models devoid of microorganisms (germ‐free/axenic models) providing insight into the host processes regulated by the presence and/or composition of the gut microbiota in health and disease (Reyniers, 1959; Smith *et al*., 2007). Based on their interaction with the host, members of the microbiota are loosely classified as beneficial/commensal species [including ‘probiotic’ bacteria, such as *Bifidobacterium* (Fanning *et al*., 2012), and benign organisms such as members of the defined “altered Shaedler flora” (Biggs *et al*., 2017)], or pathogenic species, including pathobionts such as *Helicobacter pylori* (Marshall, 2002) and opportunistic pathogens.

Those bacteria which initially colonise neonatal guts establish a mutualistic relationship with the gastrointestinal tract that can last a lifetime (Human Microbiome Project C, 2012). The birthing process has been reported to influence the type of bacteria that first colonise the infant gut; as infants acquire bacteria either by vertical transmission from the mother through the vaginal canal, and/or their environment (including the mother's skin) after caesarean section. Vaginal delivery results in the gut colonisation by pioneer bacteria including *Streptococcus*, *Escherichia*, and *Klebsiella*, which grow and establish a favourable environment (i.e., by reducing oxygen levels) for other anaerobic species including *Bifidobacterium, Lactobacillus* and *Bacteroides*, which dominate the infant gut (Houghteling and Walker, 2015). In infants born by caesarean section, early life gut microbiota tends to mimic the skin (dominated by *Staphylococcus*) and other environmental bacteria (Rutayisire *et al*., 2016). Recent research suggests maternal vaginal swabs used to inoculate caesarean delivered infants can partially restore the levels of *Bifidobacterium, Lactobacillus* and *Bacteroides* to those observed in vaginally delivered babies (Dominguez‐Bello *et al*., 2016) although longer term effects on microbial composition and host health are not yet known.

The infant gut microbial community is characterised by low diversity with high instability and is susceptible to modification by exogenous factors such as antimicrobial drugs and/or diet. Disturbances during microbiota establishment and development, for instance by antibiotic use (both through breast feeding and postnatally), can have long lasting effects on microbial composition by selecting for resistant species (Mathew, 2004; Jernberg *et al*., 2010). Infant diet also affects which type of bacteria establish the early life microbiota (Lee *et al*., 2015). In infants that are solely breast‐fed, the microbiota is simple in structure, and is dominated (upwards of 80% of the total microbiota composition) by *Bifidobacterium*, a beneficial bacterium that is often associated with probiotic traits (Serafini *et al*., 2012). Formula fed infants have a much more complex microbiota composition with significantly increased levels of *Enterobacteriaceae* and reduced levels of *Bifidobacterium* (Harmsen *et al*., 2000; Rinne *et al*., 2005; Praveen *et al*., 2015). Differences in microbial composition induced during the early development of the gut microbiota, and their longer‐term effects on host health and immunity, are likely to be affected by when, where and which species colonise the gastrointestinal tract during development.

## The host luminal environment determines the spatial distribution of a dynamic microbial community along the gastrointestinal tract

Microbial density and composition varies dramatically throughout the gastrointestinal tract and this spatial distribution seems to be independent of the mode of colonisation (e.g., co‐housing versus oral gavage in germ‐free mice), suggesting that host‐microbe interactions are the strongest determining factor for the microbial colonisation of each region (Seedorf *et al*., 2014). The gastrointestinal tract comprises a series of connected specialised segments with different environmental pressures, which affect bacterial colonisation. Below we describe the main conditions affecting the microbiota progressing along the gastrointestinal tract.

The highly acidic and enzymatic environment of the stomach, in combination with the detection of very low levels of culturable bacteria, previously led to the assumption that the stomach was a somewhat sterile environment. It is now known that the stomach harbours a highly diverse bacterial community, with Proteobacteria, Firmicutes, Bacteroidetes, Fusobacteria and Actinobacteria as the most abundant phyla, and colonisation densities ranging from 10^1^ to 10^3^ bacteria/g of content (Bik *et al*., 2006; Sheh and Fox, 2013). This diversity in the gastric microbiome is however drastically reduced in individuals harbouring *H*. *pylori*, the strongest known risk factor for developing gastric adenocarcinoma (Cho and Blaser, 2012), which is present in 50% of the human population (Brown, 2000).

**Figure 1:**
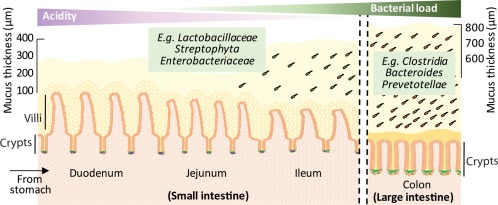
The surface of the intestinal epithelium is covered by two layers of mucus of varying thickness throughout the gastrointestinal tract. The outer, looser layer is colonised with bacteria, some of which use mucin as a food source. This layer is thickest in the colon, which contains the highest bacterial load. The inner layer is more firmly attached to the epithelium. In the colon, this layer is effectively sterile, whereas in the small intestine it has been proposed this inner layer may be penetrable to some bacteria.

Microscopic finger like projections into the intestinal lumen, termed villi, increase the surface area for absorption in the small intestine, decreasing in length from the proximal duodenum towards the distal ileum (Fig. 1). Villi are covered by a monolayer of epithelial cells comprising enterocytes, for nutrient absorption, and a variety of secretory and enteroendocrine cells. The combination of rapid transit of luminal contents and the presence of detergent‐like compounds, such as bile acids and digestion enzymes, makes the content of the proximal small intestine an unfavourable environment for bacterial colonisation (Donaldson *et al*., 2016). As a result, relatively low numbers of bacteria are found in the proximal small intestine, which tends to be dominated by a few fast‐growing facultative anaerobes and acid‐tolerant bacteria carried over from the stomach, such as Helicobacteriaceae, Streptophyta, Enterobacteriaceae and Lactobacillaceae (Seedorf *et al*., 2014). The luminal contents reaching the distal ileum form a bacterial growth‐permissive environment, comprising primarily indigestible dietary material with a pH value close to neutral, lower concentrations of compounds challenging microbial growth, and reduced oxygen availability, resulting in an increase in microbial load and complexity in the ileum as it advances towards the large intestine.

The large intestine is significantly shorter and wider than the small intestine resulting in a decreased transit rate, facilitating the epithelial absorption of large volumes of luminal fluid and permitting the establishment of a high density bacterial population of up to 10^12^–10^14^ bacteria/g of content. The majority of species belong to four distinct phyla: Proteobacteria, Actinobacteria, Bacteroides and Firmicutes (Gu *et al*., 2013; Seedorf *et al*., 2014) contributing to an extremely diverse and complex microbial environment. Ongoing work attempts to identify the many species and strains which form part of a healthy versus dysregulated or pathogenic colonic microbiota.

In addition to the increase in microbial diversity and abundance in oral‐caudal direction along the gut, there is evidence supporting differences in microbial distribution in the axial direction, from the lumen through the mucus layer toward the intestinal epithelium (Li *et al*., 2015). The mucus layer, produced by epithelial goblet cells, is present to varying degrees throughout the gastrointestinal tract but is thickest and most complex in the colon, where mucus‐producing goblet cells are more abundant (McGuckin *et al*., 2011; Juge, 2012). The mucus layer provides a barrier to protect the epithelium from direct contact with the gut content. In the colon, the mucus consists of two defined layers; a compact inner layer which is reported to be sterile, and an outer looser layer colonised with bacteria (Juge, 2012). In the small intestine, mucus is less abundant, the layers are less well defined, and may be more permeable to bacteria (Ermund *et al*., 2013). The colonic outer mucus layer is a niche that functions not only as an attachment platform, but also as a nutrient source for bacteria such as *Akkermansia muciniphila*, and members belonging to the genus such as *Lactobacilli, Bacteroides* and *Bifidobacteria* (Pretzer *et al*., 2005; Garrido *et al*., 2011; Pudlo *et al*., 2015). Bacterial selection within the mucus‐associated niche is the suggested reason for the reported detection of *Firmicutes, Lachnospiraceae* and *Ruminococcaceae* enriched communities in discrete ‘inter‐fold’ regions of the colonic mucosa and *Bacteroidetes, Prevotellaceae, Bacteroidaceae* and *Rikenellaceae* in the lumen (Nava *et al*., 2011; Donaldson *et al*., 2016).

The mucus layer is subject to a relatively high rate of turnover. For instance, the colonic outer mucus layer, together with the attached microbiota, is dislodged by peristaltic movements propelling luminal contents along the gastrointestinal tract, and is continually replenished by the inner layer produced by epithelial goblet cells (McGuckin *et al*., 2011). Recent research has highlighted the importance of metabolism and bacterial kinetics enabling *E*. *coli* and *Bacteroides thetaiotaomicron* to persist in the outer mucus layer (Li *et al*., 2015). The microbial composition of the mucus therefore results from the balance between microbial attachment and proliferation and shedding of mucus‐attached bacteria into the lumen.

## The microbiota influences gut biology

The luminal contents, and the mucus layer, condition the spatial distribution of the microbiota along the tract. In return, the rapid microbial colonisation of the infant gastrointestinal environment has profound effects on the development and functionality of host gut physiological processes and mucosal defence mechanisms (Tannock, 2007). Our current understanding of these effects owes much to a large body of *in‐vivo* work employing a range of germ‐free animal models, including mice, rats, pigs, drosophila, zebrafish, chickens and others (a number of which are summarised in Table 1). Below, we discuss interactions between gut microbes, the intestinal epithelial layer and immune and nervous systems.

**Table 1:** Summary of germ‐free phenotypes in animal models.

Feature	Altered phenotype in germ‐free	Model	References
Transit of luminal contents	Delayed gastric emptying and prolonged transit	Mouse	(Abrams and Bishop, 1967; Iwai *et al*., 1973)
		Chicken	(Palmer and Rolls, 1981; Furuse and Okumura, 1994)
Crypt‐villus morphology	Thinner villi with shallower crypts, reduced thickness of LP	Rat	(Meslin *et al*., 1973; Meslin and Sacquet, 1984)
		Mouse	(Abrams *et al*., 1963; Lesher *et al*., 1964; Glaister, 1973)
		Guinea pig	(Sprinz *et al*., 1961)
		Chicken	(Furuse and Okumura, 1994)
		Dog	(Rolls *et al*., 1978)
Epithelial microvilli	Impaired formation	Mouse	(Gordon, 1959)
Tight junctions	Increased barrier permeability	Mouse	(Smith *et al*., 2007)
Epithelial turnover	Reduced proliferation, migration and renewal in the gut	Mouse	(Khoury *et al*., 1969; Rakoff‐Nahoum *et al*., 2015)
		Rat	(Guenet *et al*., 1970)
		Pig	(Kenworthy and Allen, 1966)
		Dog	(Heneghan, 1965)
		Chicken	(Rolls *et al*., 1978)
		Zebrafish	(Bates *et al*., 2006)
	Impaired stem cell division	Drosophila	(Buchon *et al*., 2009; Cronin *et al*., 2009)
Paneth cells	Reduced in number, release of antimicrobial peptides and increased bacterial contact with epithelium	Mouse	(Cash *et al*., 2006; Yu *et al*., 2016)
		Rat	(Satoh, 1984)
Goblet cells	Reduced in number	Mouse	(Yu *et al*., 2016)
		Rat	(Gustafsson and Maunsbach, 1971)
		Chicken	(Cheled‐Shoval *et al*., 2014)
	Thin mucus layers, altered bilayer structure	Mouse	(Johansson *et al*., 2015; Li *et al*., 2015)
	Altered mucus composition	Rat	(Szentkuti and Enss, 1998)
Secondary lymphoid structures (MLN, PP, ILF, cryptopatches and spleen)	Smaller and fewer number with poorly organised structure	Mouse	(Abrams *et al*., 1963; Shroff *et al*., 1995; Yamanaka *et al*., 2003; Bouskra *et al*., 2008; Tsuji *et al*., 2008)
	Decreased neutrophil recruitment	Zebrafish	(Kanther *et al*., 2014)
T lymphocytes	Reduced in number and proportion of regulatory T cells	Mouse	(Macpherson and Harris, 2004; Geuking *et al*., 2011)
B lymphocytes	Reduced in number and mature immunoglobulin class‐switched B/plasma cells	Mouse	(Benveniste *et al*., 1971b; Benveniste *et al*., 1971a)
		Guinea pig	(Sprinz *et al*., 1961)
	Skewed isotype switching in the gut (IgA→IgE)	Mouse	(Cahenzli *et al*., 2013)
	Reduced activation of NF‐κB signalling	Zebrafish	(Kanther *et al*., 2011)
Enteric vasculature	Diminished villus capillary density and complexity	Mouse	(Stappenbeck *et al*., 2002; Reinhardt *et al*., 2012)
Enteric nervous system	Morphological and functional alteration of neurons and glia	Mouse	(Collins *et al*., 2014; McVey Neufeld *et al*., 2015)
		Rat	(Husebye *et al*., 2001)
Enteric glial cells	Reduced in number and impaired migration	Mouse	(Kabouridis and Pachnis, 2015)

### Microbial targeting of epithelial turnover

During development, the initially flat mucosa develops into the characteristic crypt‐villus architecture via a series of tissue‐patterning and cell fate determination processes coordinating the development of the epithelium and the underlying mesenchyme and smooth muscle (Walton *et al*., 2012). Ongoing maturation of crypts and villi continues through the postnatal and weaning periods in parallel with microbial colonisation of the gut, eventually culminating in mature crypts containing stem cells, Paneth cells and enterocytic/secretory precursors, and villi composed of differentiated enterocytes, goblet cells, tuft cells and a variety of enteroendocrine cells. Cell proliferation within crypts is the principal force driving cell migration on the villi (Parker *et al*., 2017) and the equilibrium of cell number and turnover is maintained by compensatory cell shedding from the villus tip into the gut lumen (Gerbe *et al*., 2011; Clevers, 2013). The continual production, migration and shedding of epithelial cells presents a dynamic barrier to microbial attachment and persistence.

**Figure 2:**
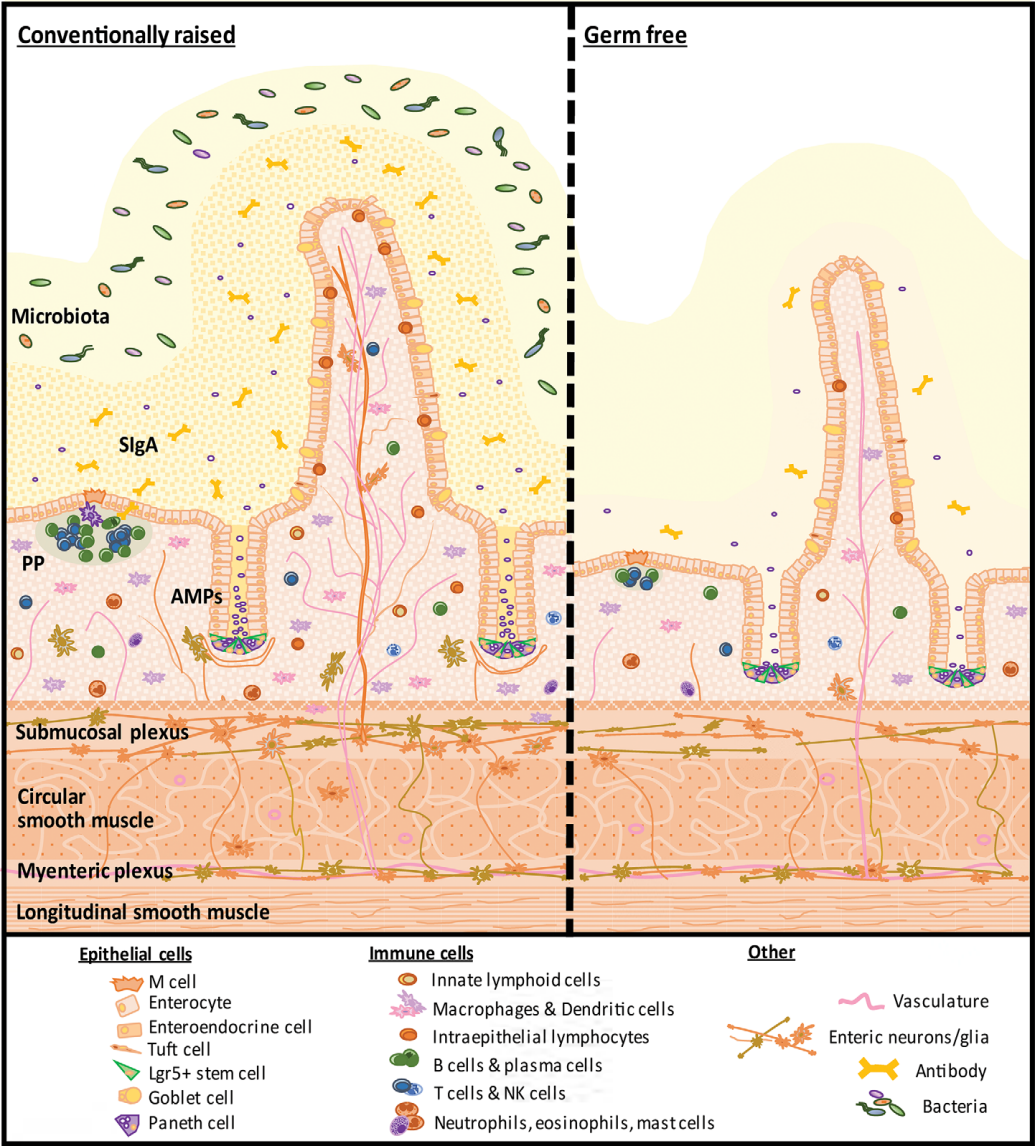
Major components of the ileal mucosa and differences between conventionally raised and germ‐free mice. The presence of microbiota influence mucus composition, crypt‐villus morphology, epithelial immune receptor expression and AMPs release, immune structure and cell composition, vascularisation, innervation, glial networks and mucosal thickness. SIgA: Secretory Immunoglobulin A, PP: Peyer's patch, AMPs: antimicrobial peptides.

The small intestines of germ‐free mice have reduced overall mass and surface area, thinner villi, shallower crypts with decreased cell proliferation and reduced migration along the crypt‐villus axis (Fig. 2) (Sommer *et al*., 2015). Likewise, in the colon of germ‐free or conventionally raised but antibiotic‐treated mice, cell proliferation rate is reduced and crypts contain fewer cells than those of conventional mice. In Drosophila, Lactobacilli can modulate gut stem cell proliferation via release of reactive oxygen species (Jones *et al*., 2013) and microbe‐induced JAK‐STAT signalling is essential for stem cell division (Buchon *et al*., 2009). Together these data suggest the presence of microbes not only promotes, but is required for normal epithelial development and turnover.

On the other hand, pathogenic bacteria can specifically inhibit epithelial turnover processes to facilitate their spread. Some bacteria specifically target cell turnover processes in order to affect the barrier integrity and persist in the epithelium, for example, by altering crypt cell gene expression programs governing cell cycle control and proliferation (Rakoff‐Nahoum *et al*., 2015; Sommer *et al*., 2015). Recent studies in mouse models and *in‐vitro* organoid models, detailed below, demonstrate that microbial signalling can alter epithelial turnover via activation of pattern recognition receptors (PRR) expressed on crypt stem cells, to alter cell proliferation and survival decisions (Neal *et al*., 2012; Hormann *et al*., 2014; Nigro *et al*., 2014). Microbial effects on epithelial turnover can also occur indirectly, by the induction of neurotransmitter and cytokine release from lamina propria cells (e.g., neuroglial, immune and stromal cells) acting on the epithelium (Hyland and Cryan, 2016; Obata and Pachnis, 2016).

**Figure 3:**
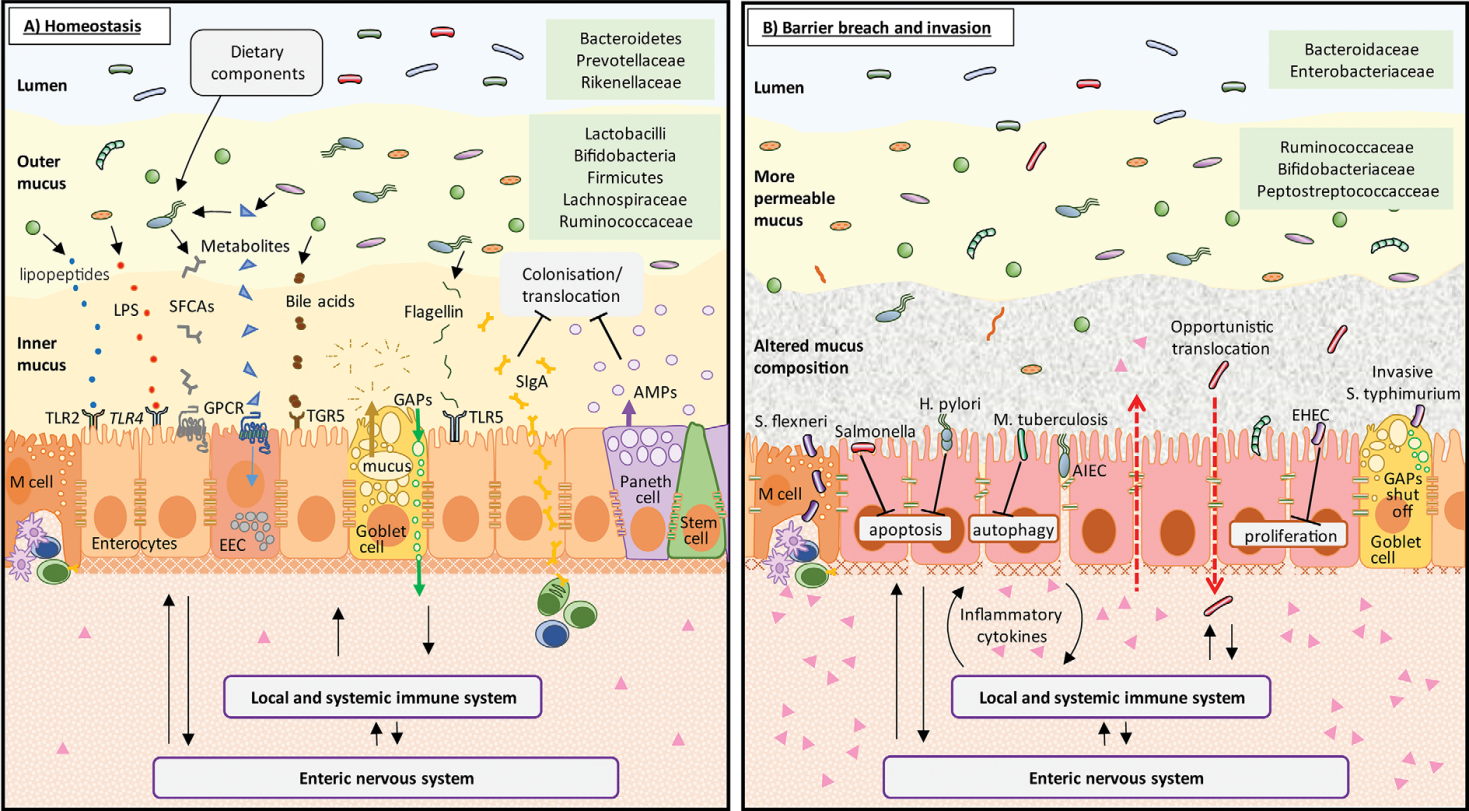
A. The intestinal epithelial barrier has multiple mechanisms for detecting and limiting bacterial invasion. Under homeostasis, release of Immunoglobulin A (IgA) and antimicrobial peptides (AMPs) into the mucus layer prevents most bacteria reaching the epithelial surface. Bacterial metabolites or peptides are bound by an array of surface receptors on epithelial cells, of which just a few examples are shown for simplicity. These include G‐protein‐coupled‐receptors (GPCRs) which bind short chain fatty acids (SCFA) and TLRs which detect lipopolysaccharide (LPS), lipoproteins and flagellin. Microbial signalling to epithelial cells can be relayed to the underlying immune and nervous systems to alter gut functions. The lamina propria is surveyed by many lymphocytes, phagocytic cells and other immune effector cells (not shown) and there is low production of inflammatory cytokines; B. Bacteria have evolved multiple methods of subverting epithelial defences and translocating to the lamina propria. Disrupted tight junctions, inflammatory signalling and epithelial cell death create gaps in the barrier allowing the entry of opportunistic bacteria, antigens and toxins from the lumen, which further amplify inflammatory responses. High levels of inflammatory cytokines, reduced mucus production and impaired antimicrobial production allow additional bacteria to reach and traverse the epithelial barrier leading to intestinal and potentially systemic infections and contributing to inflammatory bowel diseases.

Controlled cell death processes (apoptosis, necroptosis, pyroptosis) can serve to restrict microbial persistence and translocation across the epithelium (Negroni *et al*., 2015). Some bacterial species, including enterohemorrhagic *E.coli* (EHEC), *H*. *pylori and Campylobacter jejuni*, inhibit epithelial cell death, thus preserving their replication niche for longer and maximising their chance of translocating to underlying tissues (Lim *et al*., 2017; Song *et al*., 2017) (Fig. 3). In addition, intracellular autophagic pathways, which can also regulate cell death and proliferation to restrict bacterial invasion (Benjamin *et al*., 2013) can be targeted by species including *Orientia tsutsugamushi* and *Mycobacterium tuberculosis* (Shin *et al*., 2010; Choi *et al*., 2013) to facilitate epithelial penetrance.

Further physical challenges to microbial penetrance are provided by the epithelial cell brush border and tight junctions between neighbouring epithelial cells (Zihni *et al*., 2016), the formation of which is promoted by the presence of the normal microbiota [germ‐free mice have impaired brush‐border microvillus formation and increased barrier permeability (Gordon, 1959; Smith *et al*., 2007)]. Pathogenic bacteria, including *Salmonella Typhimurium* and invasive *E*. *coli* can target these defences by secreting proteases and neuro‐immune stimulatory ligands to impair brush border formation (Lhocine *et al*., 2015) and disrupt epithelial tight‐junctions (Fig. 3) (Awad *et al*., 2017; Shawki and McCole, 2017).

### Microbial targeting of epithelial immune defences

Besides the physical impairments to colonisation provided by epithelial structure and turnover, epithelial cells of all types have immune defence mechanisms to limit and/or respond to microbial invasion. Accordingly, microbes also target these mechanisms to gain access to epithelial cells and underlying tissues.

Epithelial cells express a range of immune PRR, including various Toll‐like receptors (TLR) and nucleotide‐binding oligomerization domain‐like receptors (NLRs), which survey both luminal and basolateral membranes of the barrier, and intracellular compartments. During development, alterations in epithelial expression and activity of these receptors occurs concomitantly with the morphological maturation of the epithelium and microbial colonisation and establishment. Furthermore, reduced epithelial TLR expression in germ‐free and antibiotic‐treated mice, and subsequent recovery in recolonised mice, suggests the normal microflora promote the expression of the receptor repertoire (Hormann *et al*., 2014). In neonatal mice, tuning of receptor repertoires [downregulation of the TLR4 signalling pathway (Lotz *et al*., 2006) and upregulation of TLR9 (Pott *et al*., 2012)] drives a more tolerant response to microbes. This, combined with upregulation of antiviral TLR3 expression at weaning (Pott *et al*., 2012), suggests the TLR repertoire is customised to prepare the foetal epithelium for colonisation and facilitate development of a stable microbiota, preparing the host for the uptake of new foods and antigens. In adults, triggering of PRR signalling by pathogenic bacteria, or commensal species under inflammatory conditions, promotes a range of immune responses, which limit further invasion and clear infected cells. Some species therefore inhibit PRR signalling pathways to mask their presence or prevent death of the cells they have infected (McGuire and Arthur, 2015). Bacterial triggering of TLRs can also promote the local release of pro‐inflammatory cytokines such as TNFα, which can disrupt epithelial tight junctions leading to a ‘leaky’ barrier and allowing the ingress of opportunistic bacterial species.

Paneth cells, specialised epithelial cells which appear at the base of crypts during the suckling to weaning transition, not only express PRRs but can also can release a variety of soluble antimicrobial peptides into the mucus, targeting of a vast range of organisms including gram‐positive and gram‐negative bacteria, parasites, fungi and some viruses (Kopp *et al*., 2015). Upon colonisation of the GI tract, Paneth cell release of RegIII proteins (α, β, γ) into the lumen contributes to regulating microbial composition by specifically targeting Gram‐positive bacteria (Cash *et al*., 2006). In the absence of RegIII‐γ there is increased bacterial contact with the epithelium and an increased adaptive immune response against commensals (Vaishnava *et al*., 2011). Not surprisingly, some bacteria have devised counter strategies to subvert these antimicrobial peptides. *Helicobacter pylori* for example exploits host cholesterol to obtain resistance to the antimicrobial peptide LL‐37 in the gerbil intestine (McGee *et al*., 2011) and can also selectively inhibit human beta defensin 3 (hDB3) (Bauer *et al*., 2013).

In addition to producing mucus, goblet cells have been reported to deliver antigens across the epithelium via so‐called goblet cell associated antigen passages (GAPs) (McDole *et al*., 2012; Knoop *et al*., 2015). Deletion of goblet cells in a mouse model of wild type *Salmonella* Typhimurium infection prevented the translocation bacteria to the draining mesenteric lymph nodes, indicating that *S*. Typhimurium uses goblet cells as an entry portal. Furthermore, luminal exposure to an *invasive S*. Typhimurium, shut off GAP‐associated translocation suggesting that goblet cell sensing of an invasive pathogen to shut off GAPs is a host defence mechanism (Kulkarni *et al*., 2016; Knoop *et al*., 2017).

Additional specialised epithelial cells, ‘microfold’ or ‘M’‐cells, located in the follicle‐associated epithelium overlying Peyer's patches and isolated lymphoid follicles, have shortened microvilli and altered extracellular matrix, allowing uptake and shuttling of antigens to innate and adaptive immune cells of the lamina propria and gut‐associated lymphoid tissues (Mabbott *et al*., 2013) (Fig. 3). Pathogenic bacteria including *S*. Typhimurium and *Shigella flexneri* target M cells as sites of entry, via intracellular trafficking mechanisms, by specifically killing the M cells to create an entry portal, or more generally by inducing a local inflammatory immune response to create a leaky epithelium (Jones *et al*., 1994; Corr *et al*., 2008).

### Microbial interaction with the mucosal immune system

Upon colonisation of the intestine, bacteria and bacterial products (including lipopolysaccharides, DNA, RNA, flagellin, etc.) are recognised by receptors including TLR and NLR not only on epithelial cells, but also on cells of the mucosal immune system including monocytes, macrophages, granulocytes, B cells, natural killer cells and dendritic cells, which direct appropriate pro‐inflammatory or tolerogenic immune responses (Ausubel, 2005; Hooper and Macpherson, 2010). Intestinal dendritic cells (DCs) residing in the lamina propria and Peyer's patches have been suggested to sample bacterial antigen in the lumen through a variety of methods. These include possible extension of transepithelial dendrites (Rescigno *et al*., 2001; Farache *et al*., 2013) and interception of antigens transcytosed antigens across epithelial goblet cells and M cells as described above. The presence of bacterial antigens in the lamina propria and Payer's patches contributes to the development and maturation of the adaptive immune system and the epithelium, including the development of M cells positioned over PP in the follicle‐associated epithelium. Integrated within the epithelium, a subset of innate lymphoid cells (ILCs) can also be activated by microbial stimuli to produce pro‐ or anti‐inflammatory cytokines. Recent work suggests particular cell subsets (NKp46^+^ ILC3s and F4/80^+^CD11c^+^ mononuclear cells) may be conditioned by maternal microbiota during gestation, such that offspring have a reduced inflammatory immune response to the adult gut microbiota compared to offspring of germ‐free animals (Gomez de Aguero *et al*., 2016).

In the absence of a microbiota, the gastrointestinal tract is characterized by sparse infiltration of lymphocytes in the intestinal epithelial layer and lamina propria, that is particularly noticeable by the reduced number of Immunoglobulin A^+^ (IgA^+^) positive plasma cells (Benveniste *et al*., 1971a,b) and CD4^+^ T regulatory cells (Geuking *et al*., 2011) (Fig. 2). Upon colonisation, the lamina propria, Peyer's patches and mesenteric lymph nodes are infiltrated with B‐ and T‐lymphocytes, and characterised by high levels of the non‐inflammatory IgA+ plasma cells (Benveniste *et al*., 1971b; Geuking *et al*., 2011; Hooper *et al*., 2012). This expansion causes both an increase in size and number of hypoplastic B cell follicles and germinal centres in the mesenteric lymph nodes and Peyer's patches (Yamanaka *et al*., 2003); indicating further immune maturation due to colonisation. In addition, bacterial colonisation of the large intestine also leads to the maturation of cryptopatches into isolated lymphoid follicles (Shroff *et al*., 1995; Tsuji *et al*., 2008). This process appears to be mediated by the presence of Gram negative bacteria, through direct bacterial antigens interacting with NLR (specifically NOD1) signalling on host cells (Hasegawa *et al*., 2006; Bouskra *et al*., 2008) suggesting a direct link between immune development and bacterial composition in the gut. Systemically, the immune system of germ free mice has low levels of isotype‐switched immunoglobulins, as well as poor structural organisation within secondary lymphoid organs that are all reversible by colonisation (Hooper *et al*., 2012).

### Effect of the microbiota on the enteric nervous system

The enteric nervous system (ENS) comprises complex networks of neurons and glial cells involved in complex cross‐talk with immune, bacterial and epithelial cells. Under the epithelial layer, the intestinal mucosa is densely vascularised and innervated, with neuronal fibres in close proximity to, and in some cases direct contact with (Bohorquez *et al*., 2014), overlying epithelial cells and surrounded by other mesenchymal and immune cells. Together, the vascular capillary network and the neuroglial networks of the ENS regulate many aspects of homeostatic gut function, including motility, permeability, secretion and absorption (Schemann and Neunlist, 2004; Van Landeghem *et al*., 2009), and are also involved in coordinating responses during damage and repair (Veiga‐Fernandes and Pachnis, 2017). While the generation and early patterning of the vasculature and ENS takes place during embryogenesis in parallel with epithelial development (Hatch and Mukouyama, 2015), further morphological and functional maturation of these systems is profoundly influenced by the microbiota, both in early life (Stappenbeck *et al*., 2002; Kabouridis and Pachnis, 2015; Rakoff‐Nahoum *et al*., 2015) and in adulthood (Laranjeira *et al*., 2011; Reinhardt *et al*., 2012; Kabouridis and Pachnis, 2015). Germ‐free mice for example have diminished capillary density and complexity (Stappenbeck *et al*., 2002; Reinhardt *et al*., 2012), altered neuronal patterning and composition (Collins *et al*., 2014; McVey Neufeld *et al*., 2015) and impaired glial cell development and migration (Young *et al*., 2003; Kabouridis and Pachnis, 2015) (Table 1 and Fig. 2).

Commensal and pathogenic microbes can alter ENS functions by electrical signalling (Kunze *et al*., 2009), release of neurotoxins (Yang and Chiu, 2017) or by the release of neurotransmitters including serotonin, neurotrophic factor, acetylcholine and nitric oxide (Sobko *et al*., 2006; Carabotti *et al*., 2015). Bacterial proteases or TLR ligands can also modulate neural and glial cell functions by signalling through protease‐activated receptors and TLR expressed on cells of the ENS (Brun *et al*., 2013; Burgueno *et al*., 2016). Bacterial metabolites, such as short‐chain fatty acids (SCFAs) can also stimulate the local sympathetic nervous system directly via neuronal G‐protein coupled receptors (GPCR) (Kimura *et al*., 2011) or indirectly, via intriguing epithelial‐neuro‐immune cellular networks. For example, SCFA binding to enteroendocrine cells may trigger a signalling pathway involving pseudopods, neurons and glia to alter adjacent immune cell activity and gut motility (Sommer *et al*., 2015; Obata and Pachnis, 2016).

## *In‐vitro* techniques to study microbial‐gut interactions

The specific niches which exist throughout the gastrointestinal tract are largely dictated by diet/nutrients and host‐derived factors. Disentangling how different species colonise and modify these niches can be difficult in previously colonised animal models, in which “colonisation resistance” prevents the establishment of new bacterial strains, unless the resident gut microbiota is first depleted with antibiotics (Stecher *et al*., 2013). Germ‐free animal models have alterations in intestinal morphology, immunity, and physiology, and do not reproduce a natural colonisation model. To circumvent some of these issues, and as an alternative or complement to the use of animal experiments, a multitude of *in‐vitro* techniques have been established including continuous culture systems, the generation of intestinal tissue cell lines and organoids from intestinal explants, and mock community analysis *in‐silico* to study host‐microbe interactions. Many of these systems, such as continuous culture systems, can replicate flow dynamics and the microbial and physicochemical characteristics of the luminal content in the proximal and distal colon of a variety of human and animal models (Macfarlane *et al*., 1998), and are extensively used to understand microbial effects on the host, or how specific aspects of the host immune system affect microbial dynamics.

The development of intestinal epithelium *ex‐vivo* culture techniques (Sato *et al*., 2009) has provided an *in‐vitro* system to study the effect of the microbiota on stem and other crypt cells. Colonoids and small intestinal enteroids can be generated from primary tissues, biopsies and adult and induced pluripotent stem cells (iPSCs) from humans, mice and other species to form self‐organising 3D cultures containing multiple differentiated epithelial cell types which recapitulate many functions of the original organ (Spence *et al*., 2011; Clevers, 2016). Organoids can be subjected to lineage tracing, live imaging, genetic engineering, e.g. by CRISPR‐Cas9 or viral methods, drug screening, co‐culture and infection studies (Schwank *et al*., 2013; Maru *et al*., 2016). Organoid technology has been used to study microbial interaction with stem cells, detailing stem cell responses to bacterial products (Neal *et al*., 2012; Nigro *et al*., 2014). This technology can be used to probe host‐microbe interactions relating to different regions of the intestinal tract, as intriguingly, organoids generated from distinct regions of the intestine appear to retain transcriptional and functional similarities with their site of origin (Basak *et al*., 2017). For example, application of LPS reduced proliferation and increased apoptosis in ileal crypts (Neal *et al*., 2012) while there was no such response in jejunal crypts (Davies *et al*., 2015). Similar site‐specific responses to LPS are seen in *in‐vivo* models (Pritts *et al*., 2000) and may reflect the expression of different TLR repertoires in different regions of the intestine (Gourbeyre *et al*., 2015).

**Figure 4:**
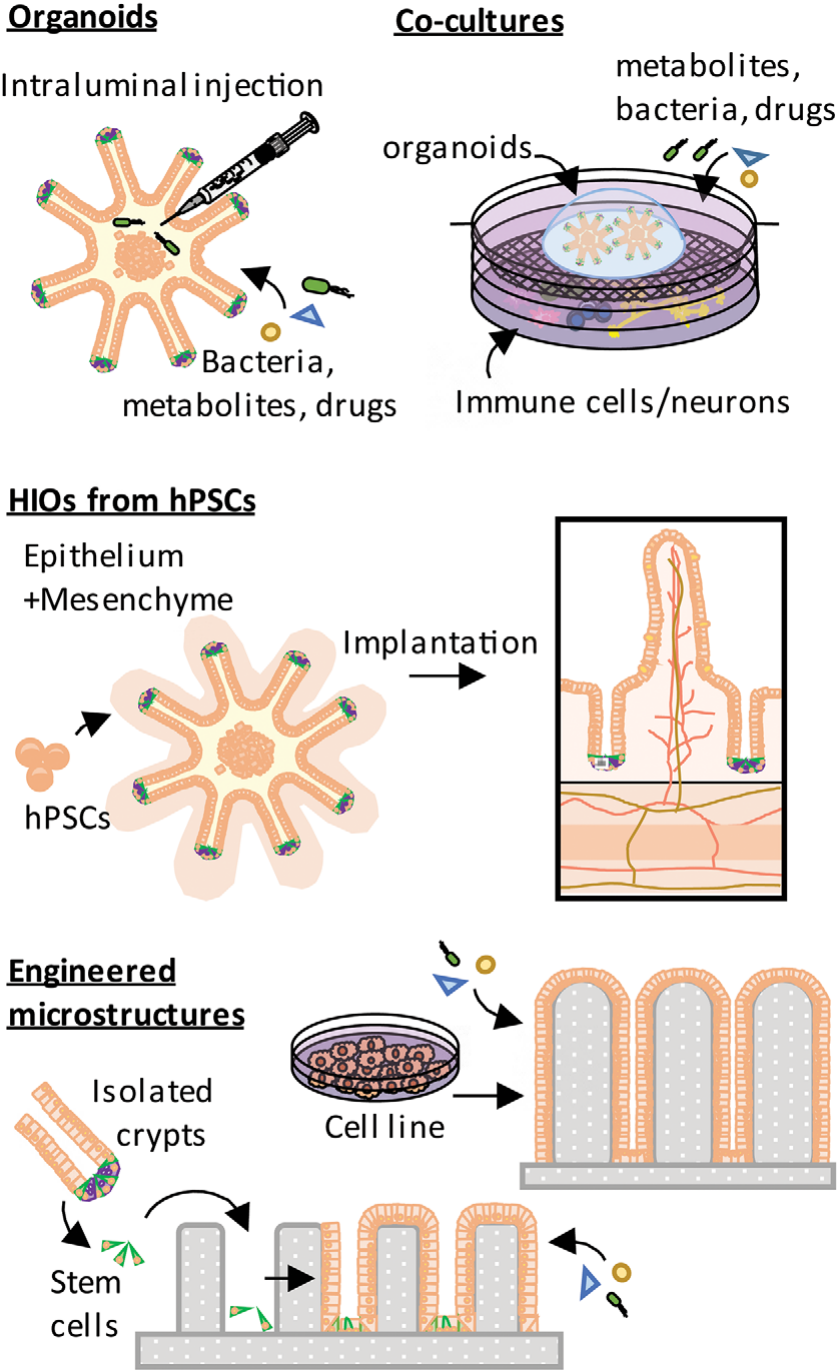
Examples of *in vitro* technologies for studying microbial interaction with epithelium and other cells of the intestine. HIOs: human intestinal organoids, hPSCs: human pluripotent stem cells

One disadvantage for the use of organoids in infection studies with bacteria is that they form closed structures where the luminal side of the epithelium is not readily accessible (Fig. 4). Recent work using luminal microinjection techniques has allowed interrogation of epithelial responses to *S*. Typhimurium species (Wilson *et al*., 2015) in small intestinal enteroids and *H. pylori* in gastric organoids (Wroblewski *et al*., 2015). Organoids can also be co‐cultured with other host cell types including myofibroblasts (Hirokawa *et al*., 2014), enteric neurons (PastuBa *et al*., 2015), intraepithelial lymphocytes (Rogoz *et al*., 2015), and macrophages (Noel *et al*., 2017) allowing studies of cross talk between the microbiota and multiple, but specific, combinations of host cell types simultaneously. Going further, multi‐tissue units have been generated from human iPSCs by the joint development of epithelial, mesenchymal and neural‐muscular layers which resulted in gut‐like units with multiple functionality (Fig. 4) (Workman *et al*., 2017) facilitating investigation of microbe‐epithelial‐neuronal interactions.

A further limitation of organoids is that they fail to replicate the crypt‐villus architecture of the small intestine and do not exhibit the characteristic steady state of the epithelium under continuous cell renewal, instead undergoing successive cycles of cyst formation, crypt enlargement and cyst bursting (Sato *et al*., 2009; Clevers, 2016). In an attempt to circumvent this issue, laser UV lithography can be used to generate patterned templates resembling the intestinal microarchitecture (Fink *et al*., 2007). The derived silicone microstructures act as scaffolds to support the proliferation of seeded immortalized cell lines, such as colonic Caco‐2 cells (Kim and Wu, 2012; Costello *et al*., 2014), although these fail to reflect many other aspects of the intestinal epithelial niche. More encouragingly, intestinal stem cells seeded on crypt patterned silicone microstructures can give rise to epithelium and mimic cell proliferation migration and shedding along the microstructure (Wang *et al*., 2014) (Fig. 4). In these models, the basal cell side in contact with the silicone is not accessible for studies on transport across the epithelial barrier or epithelial cell interactions with the vascular, nervous or immune systems, however this may be resolved by using micro‐patterned collagen porous scaffolds with accessible luminal and basal cell sides (Wang *et al*., 2017).

## Mathematical and computational modelling to study microbial‐gut interactions

The application of mathematical and computational techniques to study biological systems has enabled the study of dynamics and complex organisations and, in doing so, has often provided a mechanistic understanding of these systems not achievable by other means. Experimental information on the microbiome emerges from state‐of‐the‐art high‐throughput methods, such as metagenomics, metatranscriptomics and metabolomics. After optimal bioinformatics analysis, the resulting data enable the quantification of species abundance within the DNA sequences and viability with RNA sequencing (Heinken and Thiele, 2015). The analysis of network topology has proven useful to study the complex organisation of microbial communities (Stelling, 2004) while the integration of flux balance analyses and metagenomic studies has succeeded in reporting the metabolic activity of whole microbial communities (Klitgord and Segre, 2011). The study of host‐microbe metabolic interactions increases remarkably the complexity of network‐based models and the computational demand on flux simulations. Global human‐microbe metabolite networks have been reconstructed to describe dietary input and metabolic exchange between host and microbes (Heinken *et al*., 2013). While flux balance analysis assumes that the system is in a steady state, dynamic models comprising similar differential equations, combined with network models, can be used to predict the temporal (transient) dynamics of the microbial population across colonic compartments (Munoz‐Tamayo *et al*., 2011) or continuous spatial frameworks (Moorthy *et al*., 2015) that emerge in response to changes of the gut environment. For instance, these models have been instrumental in demonstrating that microbiota‐epithelial‐metabolic interactions have a greater impact on microbial selection than luminal compounds, which preferentially affect microbial cells that are eventually shed into the lumen (Schluter and Foster, 2012).

The term ‘host‐microbiota interactome’ was coined, under a broader vision, to describe molecular and physical interactions between the microbiota and the host, considering not only the metabolic but also the antimicrobial activity of the epithelium and mucosal immune system. The analysis of this type of system dynamics has been carried out using computational modelling techniques (Christley *et al*., 2015). Such computational models have been used to describe the spatiotemporal interactions between pathogens, T cells, macrophages, dendritic cells and epithelial cells during infection (Wendelsdorf *et al*., 2012; Alam *et al*., 2015). Simulations of experimentally inaccessible scenarios have for instance predicted that the removal of neutrophils and epithelial‐derived anti‐microbial compounds would enhance commensal bacteria growth and promote recovery against *Clostridium difficile* infection (Leber *et al*., 2015). Systems of ordinary (Arciero *et al*., 2013) and partial differential equations (Barber *et al*., 2013) have been proposed to model the epithelial and inflammatory responses to the microbiota driving the progression of necrotising enterocolitis in premature infants.

Most developed models for the microbiota do not account for the spatiotemporal cell dynamics of intestinal epithelial turnover. Analytical models have been used to describe temporal cell dynamics across separated cell compartments in the crypt‐villus axis (Johnston *et al*., 2007; Parker *et al*., 2017) and in a continuous spatial framework (Maclaren *et al*., 2017) to gain qualitative insights into the population‐level mechanisms underlying epithelial homeostasis and carcinogenesis. In the intestine, stochastic modelling of monoclonal expansion of stem cells, together with Cre/LoxP‐based and other lineage tracing experimental strategies, have been instrumental in demonstrating that small intestinal epithelial stem cells are equipotent regarding their ability to occupy with their descendants the entire gland, divide symmetrically and be replaced at random according to a neutral drift pattern (Lopez‐Garcia *et al*., 2010; Snippert *et al*., 2010; Leushacke *et al*., 2016). Among computational models, individual‐based models have been widely used to describe the spatio‐temporal dynamics of single cells, biomechanical properties, cell–cell and cell–matrix interactions, cell density effects and signalling pathways within the crypts (Pitt‐Francis *et al*., 2009; Fletcher *et al*., 2012; Dunn *et al*., 2013; Pin *et al*., 2015). Both analytical and computational models of the epithelium are suitable for extensions, in alignment with data gathering, to account for interactions with the microbiome, immune system and enteric nervous system.

Currently, significant resources are directed to understand the impact of the gut microbiome on health and disease throughout all life stages and corresponding cohort studies are being planned and conducted. Modelling strategies are an invaluable tool to face the challenge of understanding long term dynamics of the microbiome–host interactions. This type of complex system can exhibit behaviours varying from stable to erratic or emergent, and is characterised by the uncertainty of the information gathered in discontinuous sampling times and individuals. Mathematical and computational tools are essential to provide an organisational framework for complex information, and to reveal the properties and dynamics of complex long‐term microbiome‐host interactions.

## Concluding remarks

Our intestinal microbiota and intestinal physiology is the result of millions of years of co‐evolution and adaptation. Animal models, particularly germ‐free animal models, have provided some understanding of the vast complexity of host‐microbe interactions and their importance for our lifelong health. Most of these studies, although descriptive, report complex interactions that depend on the spatial location along the gut and on the life stage. The underlying mechanisms of how cross talk between the luminal microbiota and host epithelial, immune and other systems are orchestrated along the gastrointestinal tract remain to be revealed. Microbiota dysbiosis is increasingly recognised as a key feature, cause, or effect, in disorders of metabolism and immunity. A deeper understanding of the host‐microbiota interactions will therefore enable the generation of strategies to gastrointestinal/metabolic pathologies such as irritable bowel syndrome and necrotising colitis, obesity and type II diabetes.

Increasing evidence also suggests microbial influences can extend beyond the gastrointestinal and metabolic disorders to regulating development and disorders of the central nervous system (CNS). Postnatal brain development is paralleled by the maturation of the gut commensal microbiota, and CNS microglial morphology, maturation and function is (reversibly) altered in GF or antibiotic‐treated mice (Erny *et al*., 2015). Recent studies have shown microbiota‐dependent modifications in brain chemistry and behaviour, and there are reported associations between gut microbiota and CNS disorders ranging from Parkinson's to autism (Rieder *et al*., 2017; Tognini, 2017), however the specific molecular mechanisms linking changes in microbes or their metabolites during postnatal development with effects on neural development and on neurological functioning later in life are at present unknown.

The development of stem‐cell technologies such as *in‐vitro* grown tissue or multi‐tissue organic units has enabled the manipulation and visualisation of interacting components and it is already accelerating our understanding of microbial‐host interactions in the gut. Moreover, the complexity and dynamic nature of these interactions demand the use of bioinformatics analysis techniques and mathematical models to fully capture the behaviour of the system. Future application and adaptations of these technologies may permit greater understanding not only host‐environment cross talk in the gut and its importance for intestinal health, but provide insight into the wider impact of microbes in regulating systemic health and disease.
